# Attitudes towards seclusion and restraint in mental health settings: findings from a large, community-based survey of consumers, carers and mental health professionals

**DOI:** 10.1017/S2045796016000585

**Published:** 2016-08-12

**Authors:** S. A. Kinner, C. Harvey, B. Hamilton, L. Brophy, C. Roper, B. McSherry, J. T. Young

**Affiliations:** 1 Griffith Criminology Institute and Menzies Health Institute Queensland, Griffith University, Brisbane, Australia; 2 Melbourne School of Population and Global Health, University of Melbourne, Australia; 3 Mater Research Institute, University of Queensland, Brisbane, Australia; 4 School of Public Health and Preventive Medicine, Monash University, Melbourne, Australia; 5 Centre for Adolescent Health, Murdoch Childrens Research Institute, Melbourne, Australia; 6 Department of Psychiatry, University of Melbourne, Australia; 7 North Western Area Mental Health Services, Melbourne, Australia; 8 Department of Nursing, University of Melbourne, Australia; 9 St Vincent's Mental Health, Melbourne, Australia; 10 Mind Australia, Melbourne, Australia; 11 Centre for Psychiatric Nursing, University of Melbourne, Australia; 12 Melbourne Social Equity Institute, University of Melbourne, Australia; 13 Centre for Health Services Research, The University of Western Australia, Perth, Australia; 14 National Drug Research Institute, Curtin University, Perth, Australia

**Keywords:** Mental health, restraint, restrictive practices, seclusion, survey

## Abstract

**Aims.:**

There are growing calls to reduce, and where possible eliminate, the use of seclusion and restraint in mental health settings, but the attitudes and beliefs of consumers, carers and mental health professionals towards these practices are not well understood. The aim of this study was to compare the attitudes of mental health service consumers, carers and mental health professionals towards seclusion and restraint in mental health settings. In particular, it aimed to explore beliefs regarding whether elimination of seclusion and restraint was desirable and possible.

**Methods.:**

In 2014, an online survey was developed and widely advertised in Australia via the National Mental Health Commission and through mental health networks. The survey adopted a mixed-methods design, including both quantitative and qualitative questions concerning participants’ demographic details, the use of seclusion and restraint in practice and their views on strategies for reducing and eliminating these practices.

**Results.:**

In total 1150 survey responses were analysed. A large majority of participants believed that seclusion and restraint practices were likely to cause harm, breach human rights, compromise trust and potentially cause or trigger past trauma. Consumers were more likely than professionals to view these practices as harmful. The vast majority of participants believed that it was both desirable and feasible to eliminate mechanical restraint. Many participants, particularly professionals, believed that seclusion and some forms of restraint were likely to produce some benefits, including increasing consumer safety, increasing the safety of staff and others and setting behavioural boundaries.

**Conclusions.:**

There was strong agreement across participant groups that the use of seclusion and restraint is harmful, breaches human rights and compromises the therapeutic relationship and trust between mental health service providers and those who experience these restrictive practices. However, some benefits were also identified, particularly by professionals. Participants had mixed views regarding the feasibility and desirability of eliminating these practices.

## Introduction

There is growing international impetus to reduce and eliminate restrictive practices in mental health settings (McSherry, 2008; McSherry & Gooding, 2013). This has arisen through consumer and carer advocacy and is increasingly supported by efforts to improve practice and policy (Huckshorn, 2004; National Mental Health Consumer and Carer Forum, 2009). The coming into force in 2008 of the United Nations Convention on the Rights of Persons with Disabilities has added further weight to initiatives targeting reducing restrictive practices (McSherry, 2008).

Seclusion and restraint are interventions, which are currently permitted for use in mental health services and other settings to control or manage a person's behaviour. Seclusion generally refers to the deliberate confinement of a person in a room or area that he or she cannot freely exit. The term ‘restraint’ may encompass the use of bodily force (physical restraint) or a device to control a person's freedom of movement (mechanical restraint) and/or the use of medication to control a person's behaviour rather than to treat a mental disorder (chemical restraint). Mental health consumers and carers in particular also use the term ‘emotional restraint’ to refer to situations where mental health consumers feel constrained from expressing their views openly and honestly to health practitioners or behaving in particular ways for fear of the consequences (National Mental Health Consumer and Carer Forum, 2009; Roper *et al.* 2015). Of all the forms of restrictive practices, emotional restraint has the least clarity and agreement in terms of definition (Roper *et al.* 2015).

The use of seclusion and restraint has been the subject of increasing attention from consumer, carer and professional bodies and policymakers. In 2010, The Royal Australian and New Zealand College of Psychiatrists stated that it was ‘committed to achieving the aim of reducing, and where possible eliminating, the use of seclusion and restraint in a way, which supports good clinical practice and provides safe and improved care for consumers’ (2010). In Australia, one of ten key recommendations made by the National Mental Health Commission (2012) was to ‘reduce the use of involuntary practices and work to eliminate seclusion and restraint’ and, in New Zealand, Te Pou Te Whakaaro Nui (the National Centre of Mental Health Research, Information and Workforce Development) has set out a framework for ‘best practice’ in the reduction and elimination of seclusion and restraint (O'Hagan *et al.* 2008).

Most literature on restrictive interventions concentrates on the reduction rather than the elimination of seclusion and/or restraint. In 2005, the National Association of State Mental Health Program Directors in the United States released a document entitled ‘Six Core Strategies to Reduce the Use of Seclusion and Restraint’ (2008). These strategies, which include leadership towards organisational change and workforce development and training, have been influential in practice. In the USA, decreases in seclusion and restraint rates have been reported from 70 facilities that have used these strategies; reductions range from 47 to 92% (LeBel *et al.* 2004; Smith *et al.* 2005; Sullivan *et al.* 2005; Barton *et al.* 2009; Lewis *et al.* 2009). In a Finnish cluster randomised controlled trial (RCT), the six core strategies were tested leading to reduction in the incidence of seclusion and restraint from 30 to 15% of total patient time in the intervention wards in a forensic hospital (Putkonen *et al.* 2013). In England, the Safewards model (Bowers, 2014) has identified aspects of working in psychiatric wards that are known to create potential ‘flashpoints’ and strategies to manage these. Safewards was subject to a cluster RCT, which indicated that acute inpatient staff can successfully intervene to manage those flashpoints to reduce conflict and use of seclusion (Bowers *et al.* 2015). This model has recently been trialled in Australia and evaluation is pending.

Little has been written about whether or how seclusion and restraint can be eliminated entirely. Only one non peer-reviewed book (Murphy & Bennington-Davis, 2005) refers to a model for eliminating the use of seclusion and restraint, and the feasibility of implementing this model in acute mental health settings has not been tested. This is despite a number of studies noting adverse consequences for those subjected to seclusion and mechanical restraint (Burns & Rose, 2013; Gerace *et al.* 2014) and concerns with human rights breaches (Kumble & McSherry, 2010).

As part of a larger study conducted by the Melbourne Social Equity Institute, University of Melbourne and funded by the Australian National Mental Health Commission, an anonymous online survey was designed to identify and assess options to reduce and eliminate seclusion and restraint, and to consider their perceived desirability and feasibility. Using a nationwide promotion and recruitment strategy, the survey targeted key personnel in hospitals, custodial and ambulatory settings, as well as individuals with lived experience, their carers, family members and support persons. In this paper we examine participants’ perspectives in relation to the benefits and harms of seclusion and restraint as well as feasibility and desirability of eliminating seclusion and restraint.

## Method

### Procedures

The survey adopted a mixed-methods design, including both quantitative multiple-choice and qualitative open-ended questions. It was promoted via email distribution lists, discussion forums, appropriate websites, the media, individual networks, relevant newsletters and stakeholders identified through the project. It was advertised via the Melbourne Social Equity Institute's website and the website of the National Mental Health Commission. The survey was hosted on the online platform SurveyMonkey^®^ and was open from 19 March to 17 May 2014. The survey received approval from the University of Melbourne's School of Population and Global Health Human Ethics Advisory Group and Health Sciences Human Ethics Sub-Committee.

### Participants

People aged 18 years or older who were interested in commenting on seclusion and restraint were invited to complete the survey. The survey was open to the general public because a wide range of people in the community has experience with mental health service provision and may have experiences with the use of seclusion and restraint.

Based on responses to a multiple-choice question about the nature of their connection to the topic, participants were categorised into three mutually exclusive groups: (1) lived experience of treatment for a mental health issue (consumers); (2) lived experience of caring for someone with a mental health issue, but no personal experience of treatment for a mental health issue (carers); and (3) experience of encountering people with mental health issues in a professional or support capacity, but no lived experience as a consumer or carer (for simplicity, referred to hereafter as professionals).

### Measures

The survey included questions about demographic characteristics, experiences related to mental illness and restrictive practices, and attitudes and beliefs regarding the use of restrictive practices in mental health settings. Development of the survey was informed by a comprehensive review of the literature, consultation with key experts in the mental health sector and guidance from consumer and carer expert advisory groups. Definitions for each form of restrictive practice (physical restraint, mechanical restraint, chemical restraint, emotional restraint and seclusion) were provided.

Emotional restraint was included in the online survey at the instigation of the Australian National Mental Health Commission's Core Reference Group and the Lived Experience Advisory Group constituted for this project. Although less well defined (Roper *et al.* 2015) and broader in scope than the other forms of restraint, the Australian Government through the Department of Social Services’ *National Framework for Reducing and Eliminating the Us*e of Restrictive Practices in the Disability Service Sector (2014) has pointed to restraint as going beyond physical, mechanical and chemical forms to involve forms of ‘power-control’ strategies. In this study the term ‘emotional restraint’ refers to situations where mental health consumers feel constrained from expressing their views openly and honestly to health practitioners or behaving in particular ways for fear of the consequences.

The perceived benefits and harms of restrictive practices (physical restraint, mechanical restraint, chemical restraint, emotional restraint and seclusion) were assessed by asking participants how likely it was that each of a list of outcomes would occur if each restrictive practice was used (1 = Never, 2 = Rarely, 3 = Often, 4 = Always). The list of potential benefits and harms was developed based on a review of the literature and in consultation with experts and consumer and carer advisory groups. Participants were also asked to indicate whether they thought it was feasible and desirable to eliminate each restrictive practice entirely. With respect to the feasibility of elimination, participants were asked: ‘In relation to the care of people experiencing mental health issues, do you believe the following can be completely eliminated from current practice?’ (yes/no). With respect to the desirability of elimination, participants were asked ‘Which of the following should be completely eliminated from current practice?’ (yes/no).

### Data management and analysis

Descriptive statistics were calculated to describe the demographic characteristics of the overall sample and of each subgroup (consumers, carers, professionals). Responses to Likert scale questions about the perceived effects of seclusion and restraint were dichotomised for the purposes of analysis into never/rarely *v.* often/always. The three benefits and the three harms endorsed by the largest proportion of participants as ‘often’ or ‘always’ occurring as a result of each restrictive practice were retained for further analysis.

We calculated the proportion of participants indicating that each benefit and harm would ‘often’ or ‘always’ occur as a result of each restrictive practice, and the proportion of participants indicating that it was desirable and feasible to eliminate each form of restrictive practice. Differences between subgroups were examined using chi-square tests.

To examine the association between lived experience and attitudes towards the desirability and feasibility of eliminating seclusion and restraint, we fitted a log-linked modified-Poisson regression as recommended previously (Zou, 2004). We calculated prevalence risk ratios (PRRs) with 95% confidence intervals (95% CI), using the ‘professional’ group (i.e., no lived experience) as the reference category. We also computed adjusted prevalence risk ratios (APRRs) adjusting for gender, age, Indigenous status, state of residency, urbanicity, and education level. Regression s.e. were adjusted for non-random survey sampling using the SVY (survey) prefix command in Stata. Because all questions were optional, the valid sample size varies between items and is shown in all Tables. All analyses were conducted using Stata version 14.1 (StataCorp, 2015).

## Results

A total of 1451 people responded to the survey. Of these, 185 responses were not analysed because the participants were aged below 18 (*n* = 4; 0.3%), residing outside of Australia (*n* = 45; 3.1%) or had not provided consent to the study (*n* = 136; 9.4%). The small number of overseas participants means that a detailed comparison of overseas and Australian participants was not appropriate. A further 78 (5.4%) responses were excluded due to missing country of residence or participant ‘exposure’ group data. We excluded responses from 38 (2.6%) participants who did not identify as either a consumer, carer or professional. The remaining 1150 (79.3%) participant responses were retained for analysis.

Table 1 presents the demographic characteristics of the sample. The majority of participants were female and had a tertiary education; relatively few participants were aged under 30 years and 21 participants (1.9%) identified as Indigenous. Although the sample included respondents from all Australian states and territories, and from urban, regional and remote areas, the majority of participants lived in a capital city (62%) and in Victoria or New South Wales (59%).

**Table 1.** Sample characteristics by group

Characteristic	Consumers *n* (%) 529 (46.0%)	Carers *n* (%) 308 (26.8%)	Professionals *n* (%) 313 (27.2%)	Total *N* (%)*	*p*-value
Female	402 (77.3%)	216 (70.8%)	217 (70.0%)	835 (73.6%)	0.041
Indigenous	11 (2.1%)	8 (2.7%)	2 (0.7%)	21 (1.9%)	0.153
Age category					<0.001
18–30 years	123 (23.3%)	33 (10.7%)	41 (13.1%)	197 (17.1%)	
31–50 years	248 (46.9%)	115 (37.3%)	143 (45.7%)	506 (44.0%)	
>50 years	158 (29.9%)	160 (52.0%)	129 (41.2%)	447 (38.9%)	
Education					<0.001
≤Year 12	79 (15.0%)	33 (10.8%)	3 (0.9%)	59 (5.2%)	
Diploma/certificate	135 (25.7%)	43 (14.0%)	40 (12.9%)	218 (19.1%)	
Tertiary education	312 (59.3%)	230 (75.2%)	268 (86.2%)	810 (70.9%)	
Remoteness					0.021
Capital city	311 (60.4%)	173 (58.0%)	208 (68.4%)	692 (61.9%)	
Regional	138 (26.8%)	95 (31.9%)	73 (24.0%)	306 (27.4%)	
Rural/remote	66 (12.8%)	30 (10.1%)	23 (7.6%)	119 (10.7%)	
State/territory					0.104
VIC	164 (32.3%)	100 (33.8%)	96 (31.8%)	360 (32.6%)	
NSW	145 (28.5%)	75 (25.3%)	76 (25.2%)	296 (26.8%)	
QLD	80 (15.8%)	35 (11.8%)	41 (13.6%)	156 (14.1%)	
SA	51 (10.0%)	40 (13.5%)	44 (14.6%)	135 (12.2%)	
WA	40 (7.9%)	20 (6.8%)	18 (6.0%)	78 (7.0%)	
TAS	5 (1.0%)	8 (2.7%)	4 (1.3%)	17 (1.5%)	
ACT	16 (3.2%)	8 (2.7%)	8 (2.7%)	32 (2.9%)	
NT	7 (1.4%)	10 (3.4%)	15 (5.0%)	32 (2.9%)	
Ever observed or affected by the seclusion and restraint of someone else for a mental health issue	408 (79.7%)	275 (89.6%)	283 (93.1%)	966 (86.0%)	<0.001

*Due to missing values, sample size ranges from 1106 to 1150.

Almost one in two participants (*n* = 529, 46%) identified as a mental health consumer; 27% (*n* = 308) identified as a carer and 27% (*n* = 313) identified as a professional. Compared with the other two groups, consumers were more likely to be female, and were on average younger and less likely to report a tertiary education. Those who identified as professionals were more likely to report a tertiary education and to live in a capital city.

### 

#### Perceived harms of the use of seclusion and restraint

The perceived harms of seclusion and restraint most frequently endorsed by participants included infringing human rights, compromising therapeutic trust, and traumatising or triggering past trauma. The majority of participants believed that all forms of restraint and seclusion would often or always cause these forms of harm. Between 80 and 90% of participants believed that all forms of restraint and seclusion infringed human rights, and with the exception of chemical restraint (74%), between 80 and 90% believed that each form of restrictive practice would often or always compromise therapeutic trust. Just under two thirds (62%) believed that chemical restraint would often or always cause trauma or trigger past trauma, while between 80 and 90% held this view for all other forms of restrictive practice.

Table 2 compares consumer, carer and professional perceptions of harms of each restrictive practice. The group least likely to believe that seclusion and restraint would cause harm was professionals, although the majority of professionals still believed that all forms of restrictive practice except for chemical restraint (49%) would often or always cause harm. In most cases the proportion of carers perceiving that such practices would cause harm was greater, and the proportion of consumers holding this belief was greater still. Just over two thirds of consumers (69%) believed that chemical restraint would often or always cause trauma or trigger past trauma.

**Table 2.** Perceived harms by group and type of restraint and seclusion

Perceived harms (valid *N*, %)	Consumers *n* (%)	Carers *n* (%)	Professionals *n* (%)	Total *N* (%)	*p*-value
Physical restraint					
Often/always infringes on human rights (*N* = 784, 68.2%)	330 (91.7%)	190 (88.8%)	178 (84.8%)	698 (89.0%)	0.039
Often/always traumatises or triggers past trauma (*N* = 813, 70.7%)	343 (91.2%)	187 (86.6%)	191 (86.4%)	721 (88.7%)	0.106
Often/always compromises therapeutic trust (*N* = 780, 67.8%)	322 (89.2%)	179 (85.2%)	166 (79.4%)	667 (85.5%)	0.006
Mechanical restraint					
Often/always infringes on human rights (*N* = 743, 64.6%)	308 (92.2%)	186 (89.9%)	176 (87.1%)	670 (90.2%)	0.156
Often/always compromises therapeutic trust (*N* = 744, 64.2%)	311 (91.7%)	180 (89.1%)	170 (83.7%)	661 (88.8%)	0.016
Often/always traumatises or triggers past trauma (*N* = 770, 66.9%)	313 (88.9%)	179 (86.9%)	182 (85.9%)	674 (87.5%)	0.536
Chemical restraint					
Often/always infringes on human rights (*N* = 771, 67.0%)	305 (86.4%)	162 (76.8%)	147 (71.0%)	614 (79.6%)	<0.001
Often/always compromises therapeutic trust (*N* = 771, 67.0%)	295 (82.9%)	148 (72.2%)	130 (61.9%)	573 (74.3%)	<0.001
Often/always traumatises or triggers past trauma (*N* = 791, 68.8%)	251 (69.0%)	130 (61.9%)	107 (49.3%)	488 (61.7%)	<0.001
Emotional restraint					
Often/always infringes on human rights (*N* = 720, 62.6%)	305 (91.0%)	174 (88.8%)	156 (82.5%)	635 (88.2%)	0.014
Often/always compromises therapeutic trust (*N* = 722, 62.8%)	307 (91.6%)	171 (86.8%)	151 (79.5%)	629 (87.1%)	<0.001
Often/always traumatises or triggers past trauma (N = 750, 65.2%)	320 (91.4%)	169 (85.3%)	148 (73.3%)	637 (84.9%)	<0.001
Seclusion					
Often/always infringes on human rights (*N* = 775, 67.4%)	323 (91.8%)	183 (85.9%)	173 (82.4%)	679 (87.6%)	0.003
Often/always traumatises or triggers past trauma (*N* = 803, 69.9%)	325 (88.3%)	179 (82.5%)	180 (82.6%)	684 (85.2%)	0.071
Often/always compromises therapeutic trust (*N* = 771, 67.0%)	313 (89.7%)	168 (80.0%)	165 (77.8%)	646 (83.8%)	<0.001

### Perceived benefits of seclusion and restraint

The perceived benefits of seclusion and restraint most frequently endorsed by participants included increasing consumer safety, increasing the safety of staff and others, and setting behavioural boundaries. Table 3 compares consumer, carer and professional perceptions of the perceived benefits of each restrictive practice. The group most likely to perceive benefits of seclusion and restraint was professionals; in most cases the proportion of carers perceiving benefits was smaller, and the proportion of consumers perceiving benefits smaller still. Nevertheless, the majority of consumers believed that physical, mechanical and chemical restraint, and seclusion, increased the safety of others. By contrast, a minority of consumers (and carers and professionals) believed that emotional restraint increased the safety of others or indeed the consumer (Table 3).

**Table 3.** Perceived benefits of restrictive practices by group

Perceived benefit (valid *N*, %)	Consumers *n* (%)	Carers *n* (%)	Professional *n* (%)	Total *N* (%)	*p*-value
Physical restraint					
Often/always increases consumer safety (*N* = 792, 68.9%)	170 (46.5%)	126 (60.9%)	158 (71.8%)	454 (57.3%)	<0.001
Often/always increases staff/others safety (*N* = 785, 68.3%)	202 (56.4%)	133 (64.9%)	160 (72.1%)	495 (63.1%)	0.001
Often/always sets behavioural boundaries (*N* = 761, 66.2%)	150 (43.9%)	110 (53.4%)	132 (62.0%)	392 (51.5%)	<0.001
Mechanical restraint					
Often/always increases consumer safety (*N* = 742, 64.5%)	132 (39.0%)	108 (54.8%)	127 (61.3%)	367 (49.5%)	<0.001
Often/always increases staff/others safety (*N* = 738, 64.2%)	211 (63.2%)	131 (66.5%)	155 (74.9%)	497 (67.3%)	0.018
Often/always sets behavioural boundaries (*N* = 722, 62.8%)	114 (35.2%)	92 (46.2%)	100 (50.2%)	306 (42.3%)	0.001
Chemical restraint					
Often/always increases consumer safety (*N* = 773, 67.2%)	195 (55.2%)	133 (65.2%)	167 (77.3%)	495 (64.0%)	<0.001
Often/always increases staff/others safety (*N* = 769, 66.9%)	236 (68.0%)	155 (76.0%)	187 (85.8%)	578 (75.2%)	<0.001
Often/always sets behavioural boundaries (*N* = 745, 64.8%)	119 (35.8%)	95 (47.0%)	104 (49.3%)	318 (42.7%)	0.003
Emotional restraint					
Often/always increases consumer safety (*N* = 713, 62.0%)	64 (19.4%)	48 (25.3%)	58 (29.9%)	170 (23.8%)	0.022
Often/always increases staff/others safety (*N* = 706, 61.4%)	103 (32.0%)	66 (34.9%)	69 (35.4%)	238 (33.7%)	0.672
Often/always sets behavioural boundaries (*N* = 697, 60.6%)	87 (27.6%)	70 (36.7%)	65 (34.0%)	222 (31.9%)	0.080
Seclusion					
Often/always increases consumer safety (*N* = 775, 67.4%)	156 (44.1%)	117 (57.1%)	154 (71.3%)	427 (55.1%)	<0.001
Often/always increases staff/others safety (*N* = 767, 66.7%)	214 (62.4%)	148 (72.2%)	187 (85.4%)	549 (71.6%)	<0.001
Often/always sets behavioural boundaries (*N* = 751, 65.3%)	150 (44.5%)	105 (52.0%)	135 (63.7%)	390 (51.9%)	<0.001

### Perceived feasibility and desirability of eliminating seclusion and restraint

Participants were asked whether they thought it was desirable or feasible to completely eliminate seclusion and restraint (Table 4). The vast majority of participants believed that it was both desirable and feasible to eliminate emotional restraint and mechanical restraint; just over half believed that it was both desirable and feasible to eliminate seclusion. By contrast, just over a third of participants believed that it was desirable to eliminate physical and chemical restraint, and around one in four believed that this was feasible. There were marked differences between groups, with consumers most likely and professionals least likely to believe that it was both desirable and feasible to eliminate such practices. The notable exception was emotional restraint, with the vast majority of participants in all groups believing that it was both desirable and feasible to eliminate this practice. Only a small minority of professionals believed that it was feasible to eliminate physical restraint (14%) or chemical restraint (13%).

**Table 4.** Perceived feasibility and desirability of eliminating seclusion and restraint, by group

Perception of elimination (valid *N*, %)	Consumers *n* (%)	Carers *n* (%)	Professionals *n* (%)	Total *N* (%)	*p*-value
Physical restraint					
Desirable (*N* = 560, 48.7%)	122 (48.8%)	53 (34.4%)	39 (25.0%)	214 (38.2%)	<0.001
Feasible (*N* = 581, 50.5%)	87 (34.1%)	31 (19.1%)	23 (14.0%)	141 (24.3%)	<0.001
Mechanical restraint (*N*, %)					
Desirable (*N* = 608, 52.9%)	223 (80.5%)	118 (72.8%)	121 (71.6%)	462 (76.0%)	0.056
Feasible (*N* = 585, 50.9%)	199 (73.4%)	96 (61.9%)	107 (67.3%)	402 (68.7%)	0.044
Chemical restraint					
Desirable (*N* = 565, 49.1%)	116 (46.4%)	53 (35.1%)	33 (20.1%)	202 (35.8%)	<0.001
Feasible (*N* = 588, 51.1%)	93 (35.2%)	37 (23.4%)	21 (12.7%)	151 (25.7%)	<0.001
Emotional restraint					
Desirable (*N* = 629, 54.6%)	276 (90.5%)	137 (87.3%)	149 (89.8%)	562 (89.5%)	0.558
Feasible (*N* = 584, 50.8%)	234 (82.7%)	114 (78.1%)	127 (81.9%)	475 (81.3%)	0.498
Seclusion					
Desirable (*N* = 575, 50.0%)	177 (66.8%)	92 (56.8%)	47 (31.8%)	316 (55.0%)	<0.001
Feasible (*N* = 590, 51.3%)	154 (56.8%)	69 (44.5%)	40 (24.4%)	327 (55.4%)	<0.001

Compared with professionals, both carers and consumers were significantly more likely to believe that elimination of chemical restraint and seclusion was both desirable and feasible. Consumers were significantly more likely than professionals to believe that elimination of physical restraint was desirable and feasible. These associations persisted after adjustment for potential confounding factors (Table 5).

**Table 5.** Association between lived experience and feasibility/desirability of elimination

	Desirable		Feasible	
	PRR (95% CI)	APRR (95% CI)*	PRR (95% CI)	APRR (95% CI)*
Physical restraint				
Professionals	1	1	1	1
Carers	1.33 (0.94–1.89)	1.32 (0.92–1.90)	1.28 (0.77–2.11)	1.24 (0.74–2.06)
Consumers	1.88 (1.39–2.54)	1.72 (1.25–2.36)	2.30 (1.51–3.49)	1.87 (1.20–2.91)
Mechanical restraint				
Professionals	1	1	1	1
Carers	1.02 (0.89–1.17)	1.05 (0.91–1.20)	0.91 (0.77–1.07)	0.93 (0.78–1.10)
Consumers	1.12 (1.00–1.26)	1.12 (0.99–1.27)	1.08 (0.95–1.23)	1.05 (0.91–1.21)
Chemical restraint				
Professionals	1	1	1	1
Carers	1.69 (1.16–2.46)	1.87 (1.25–2.79)	1.71 (1.04–2.83)	1.79 (1.06–3.02)
Consumers	2.23 (1.60–3.12)	2.25 (1.57–3.23)	2.71 (1.76–4.18)	2.13 (1.34–3.37)
Emotional restraint				
Professionals	1	1	1	1
Carers	0.98 (0.90–1.06)	0.99 (0.92–1.08)	0.96 (0.86–1.07)	0.97 (0.87–1.09)
Consumers	1.01 (0.95–1.08)	1.03 (0.95–1.10)	1.00 (0.91–1.10)	1.03 (0.93–1.14)
Seclusion				
Professionals	1	1	1	1
Carers	1.79 (1.36–2.35)	1.79 (1.35–2.36)	1.79 (1.29–2.49)	1.69 (1.21–2.34)
Consumers	2.10 (1.63–2.71)	1.89 (1.45–2.46)	2.31 (1.72–3.09)	2.00 (1.48–2.72)

PRR, prevalence risk ratio; APRR, adjusted prevalence risk ratio.

*Model adjusted for gender, age, Indigenous status, state of residency, urbanicity and education level.

## Discussion

In this national survey we found that a large majority of participants believed that seclusion and restraint were likely to cause harm, including breaching human rights, compromising therapeutic trust and potentially causing or triggering past trauma. Evidently, seclusion and restraint are practices of great concern for all stakeholders, and our findings suggest a strong preference for minimising their use in Australia, while pursuing prevention and alternative strategies. However, many participants, particularly professionals, also believed that seclusion and some forms of restraint were likely to produce some benefits, including increasing consumer safety, increasing the safety of staff and others, and setting behavioural boundaries.

While emotional restraint is generally not well defined, and rarely regulated, it appears that many participants did acknowledge this practice and had serious concerns about it. Only a minority of participants believed that this practice resulted in any benefits and the vast majority believed that it was harmful, and that it was feasible and desirable to eliminate its use. Finally, the vast majority of participants believed that it was both desirable and feasible to eliminate mechanical restraint.

There were some notable differences in the responses of participants according to whether they had lived experience. Consumers were more likely than professionals to believe that seclusion and restraint caused harm, although in all groups the vast majority identified harms associated with these practices. This widespread belief identified amongst consumers of the harm inflicted by restrictive practices echoes the findings of Burns & Rose (2013) that such coercive treatment is experienced by consumers ‘as a naked expression of their powerlessness‘ (p. 88). Conversely, professionals were more likely than consumers to identify benefits associated with restrictive practices. This may be because, for professionals, seclusion and restraint are often viewed through a lens of occupational health and safety, such that restrictive interventions are viewed as a necessary response to behaviours of concern (Chan, 2016). In both cases, the responses of carers fell between those of consumers and professionals on most questions. These findings indicate that, perhaps not surprisingly, people with lived experience are more sensitised to the harms of seclusion and restraint, and less likely to believe that there are benefits to such practices (Brophy *et al.* 2016*a*, *b*).

Notwithstanding the above, the broad agreement on harms associated with restrictive practices across the three surveyed stakeholder groups has not previously been identified. Indeed, earlier research showed strong divergence between views of staff and patients in three acute wards of one service in the USA, with staff expressing unanimous support for seclusion and little awareness of harms identified by consumers (Soliday, 1985). More recently in the Netherlands, a survey of the views of 540 psychiatric services staff indicated that nurses and psychiatrists who regularly undertook seclusion rated it as largely positive (Van Doeselaar *et al.* 2008), whereas consumer experiences of seclusion and restraint are consistently reported as negative (Steinert *et al.* 2013).

The agreement identified here between stakeholders is a helpful foundation for future practice and policy change, reflecting a more nuanced and shared understanding among key stakeholders. Shared recognition of the significant harms that can be caused by restrictive practices could inform future education, training and organisational change in Australia, building on previous international efforts to change professional attitudes (e.g., Mann-Poll *et al.* 2013) and create greater awareness. Recent Australian evidence suggests that the notion that restrictive practices are a ‘necessary evil’ may be modifiable as a consequence of staff training (Mann-Poll *et al.* 2013, p. 4). Our findings may be indicative of an increased willingness among practitioners to change their use of restrictive practices; an enhanced appreciation of how harmful restrictive practices have been for some consumers may enable further support for those seeking change. An overview of strategies that show promise in reducing the use of seclusion and restraint has been provided by the research team in its report to Australia's National Mental Health Commission (Melbourne Social Equity Institute, 2014). Incorporation of consumer and carer perspectives in staff training could be particularly beneficial in terms of enabling shared recognition of the significant harms experienced (Huckshorn, 2004). If the perceived benefits of restrictive practices are overshadowed by concerns about corrupting the potential for inpatient services to be therapeutic environments, being complicit in human rights breaches and longer term harm associated with trauma, then working towards elimination may become more feasible. Chan (2016) has pointed out that staff safety and human rights are not mutually exclusive and positive behaviour support can be used as a preventive strategy to avoid the use of restrictive interventions. Programs for reducing these practices have previously recognised the need to intervene systemically rather than solely focussing on interventions with individual consumers and others have operationalised a multilevel approach to change (Huckshorn, 2004; Bowers, 2014).

### Strengths and limitations

This is the first ever national Australian survey of beliefs and attitudes regarding seclusion and restraint and, to our knowledge, the only one that has asked the same questions of the three important stakeholder groups. It comes at a time when there are increasing calls to reduce and where possible eliminate these practices in mental health settings. Key strengths of the study include the large, national sample including consumers, carers and professionals, and efforts to bring nuance to the debate by considering perceived harms and benefits, and giving separate consideration to the desirability and feasibility of elimination.

One important limitation of the study was convenience sampling, with the sample skewed towards urban settings and the most populous states of Australia. However, study analyses were adjusted for urbanicity and non-random survey sampling. Members of the professional group were not asked to specify whether they had participated in or overseen the use of seclusion and restraint or whether they worked in general with consumers and carers. An analysis of these sub-groups would have offered useful comparative data. Analyses were confined to responses from Australian participants and it may be that beliefs and attitudes differ in other countries. Participants were by definition sufficiently motivated to complete the voluntary survey, and as a result, individuals with strong views regarding seclusion and restraint were probably over-sampled. Another limitation is that despite providing participants with clear definitions of seclusion and each form of restraint, their responses cannot be guaranteed to reflect these definitions. Finally, this study did not examine attitudes towards restrictive practices in specific contexts or settings (e.g., emergency departments, prisons), despite that elimination of such practices is likely to be more easily achieved in some settings than others. Exploration of setting-specific attitudes to seclusion and restraint would be a fruitful avenue for future research.

### Conclusion

A national survey of consumers, carers and professionals indicated differences in attitudes about whether or not seclusion and restraint can be eliminated. However, there was strong agreement that the use of seclusion and restraint can be harmful because of breaches to human rights, compromising therapeutic relationships and trust and people experiencing trauma or being re-traumatised. On the other hand, some benefits were identified and there were differences of opinion about the desirability and feasibility of elimination of these practices. In the case of emotional restraint, there was consensus that it both could and should be eliminated from current practice. These findings are a resource to the field for the continued work of reducing restrictive practices.

## Availability of Data and Materials

Access to the data is subject to multiple ethics approvals, and as such the authors do not have the authority to grant access. Individuals wishing to access the data should contact Stuart Kinner (s.kinner@unimelb.edu.au) in the first instance.
